# Supplementary material for: Metabolome and Transcriptome Profiling Unveil the Mechanisms of Polyphenol Synthesis in the Developing Endopleura of Walnut (Juglans regia L.)
Source: Int J Mol Sci. 2022 Jun 14;23(12):6623. doi: 10.3390/ijms23126623 (PMC9224426; doi:10.3390/ijms23126623)
Supplement: Supplementary file 1 [file ijms-23-06623-s001.zip › ijms-1741232-supplementary/Supplementary Figures S1-S8.pdf]

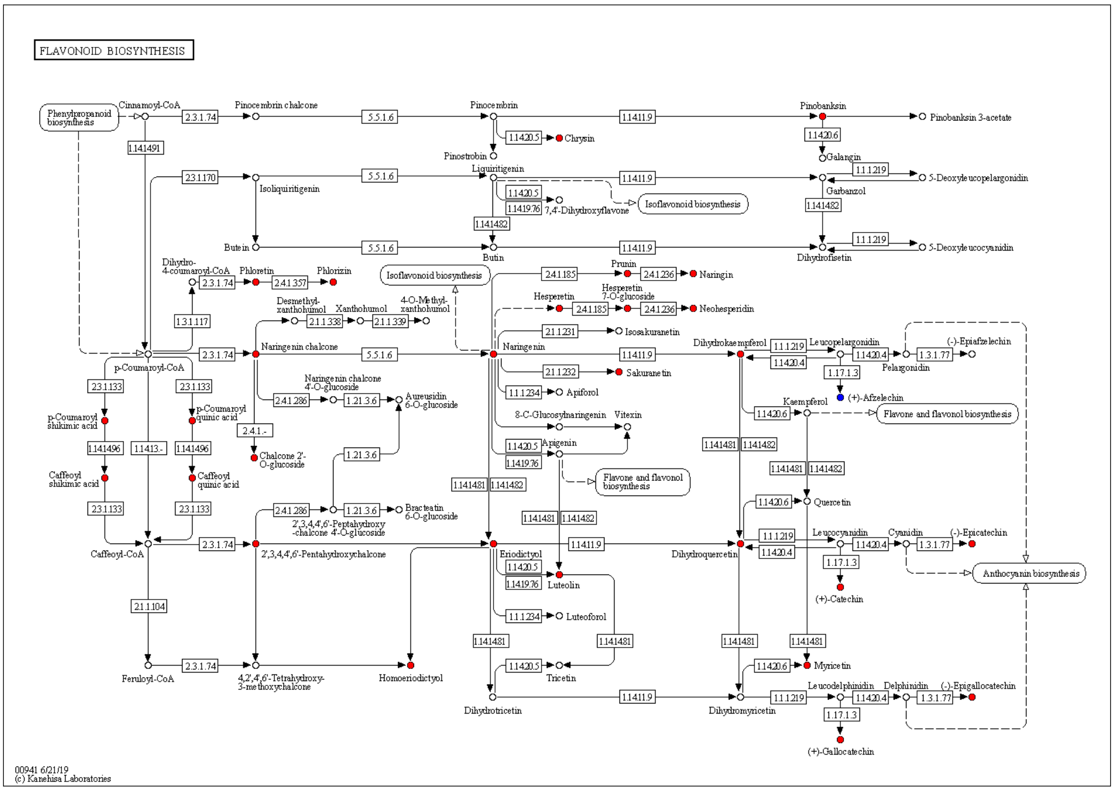

Figure S1. Flavonoid biosynthesis analysis of the DAMs between the endopleura and embryo. Red metabolites indicate high accumulation in the endopleura, green metabolites indicate high accumulation in the embryo, and blue metabolites indicate no difference between the endopleura and embryo.

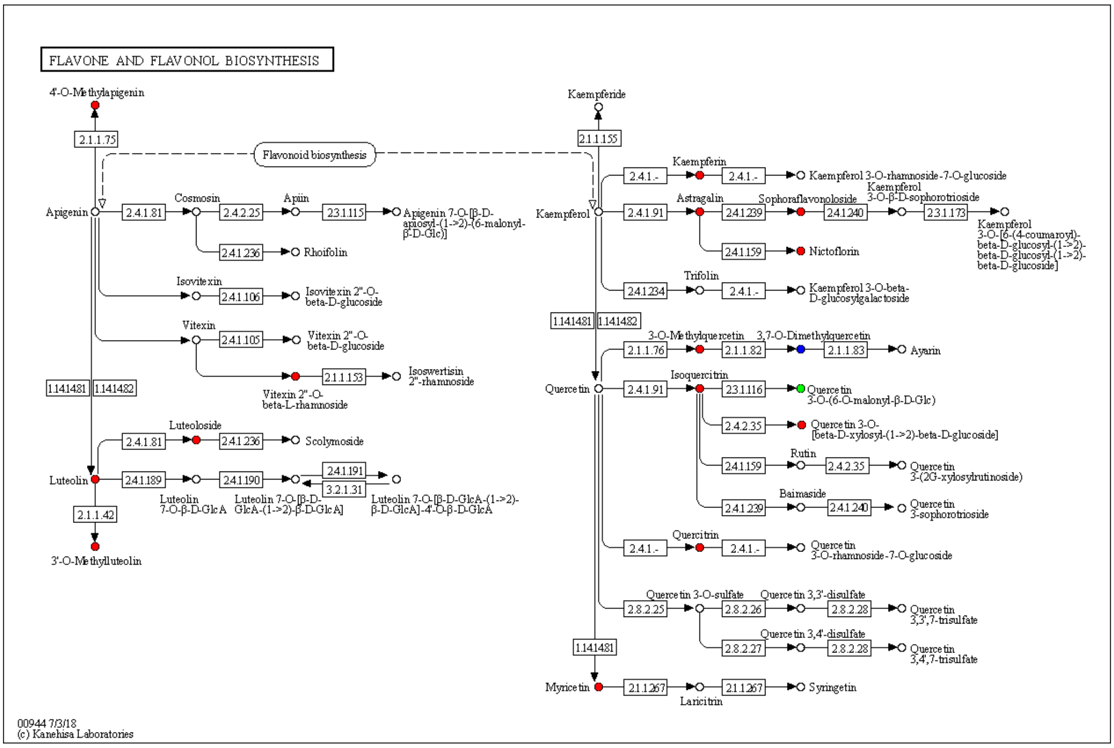

Figure S2. Flavone and flavonol biosynthesis analysis of the DAMs between the endopleura and embryo. Red metabolites indicate high accumulation in the endopleura, green metabolites indicate high accumulation in the embryo, and blue metabolites indicate no difference between the endopleura and embryo.

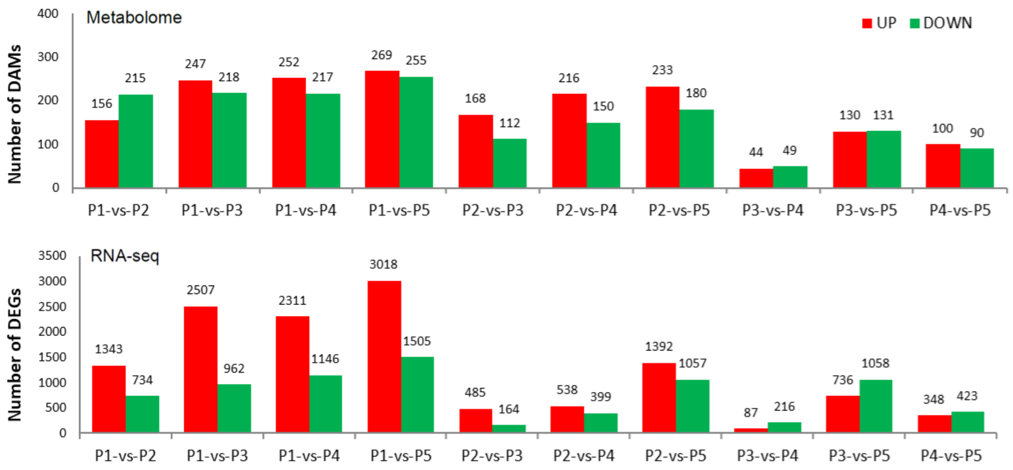

Figure S3. Pairwise comparison between samples at each stage in endopleura. A total of 632 DAMs and 6300 DEGs were identified.

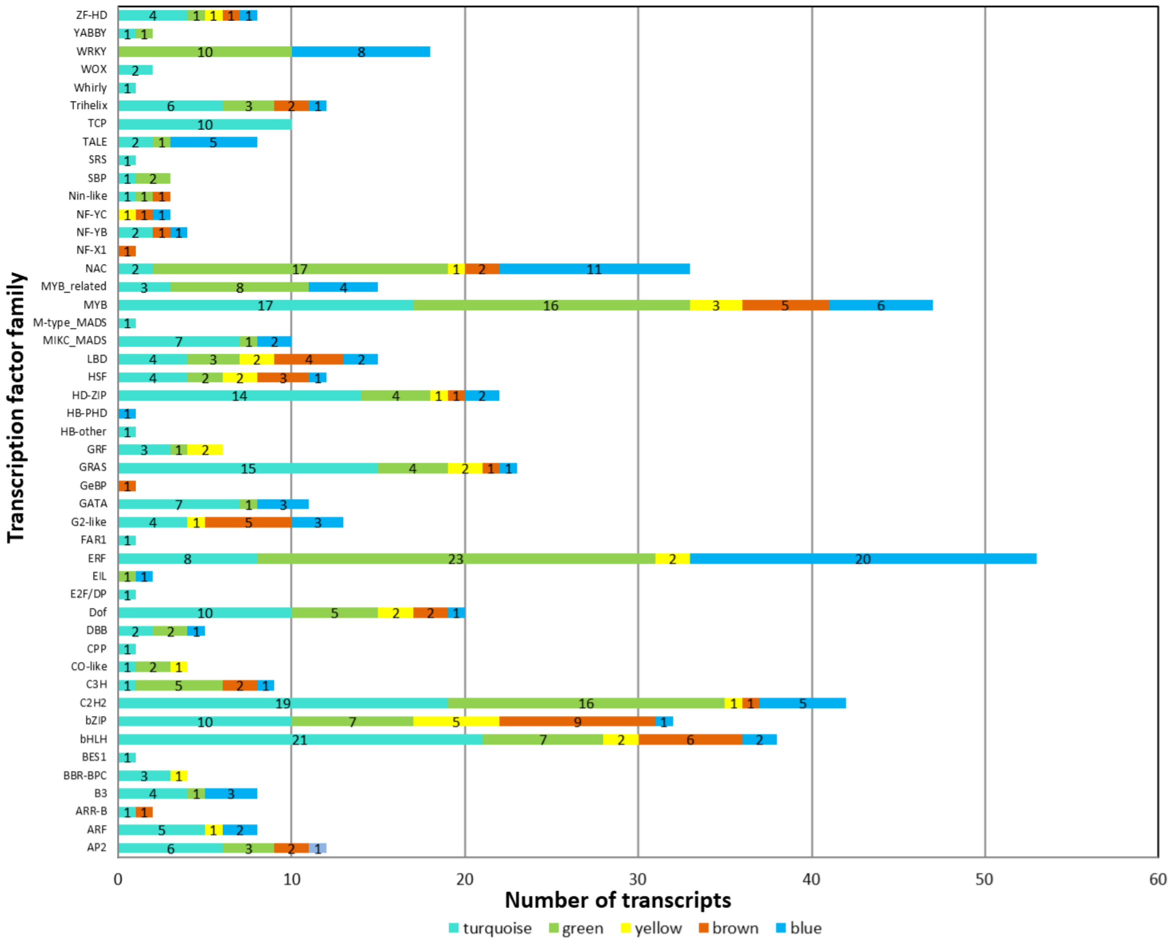

Figure S4. Transcription factors in moudels. Transcription factors in the developing walnut endopleura and embryo. In total 529 DEGs were predicted to be transcription factors and belonged to 47 families. The five color modules were from Figure 5.

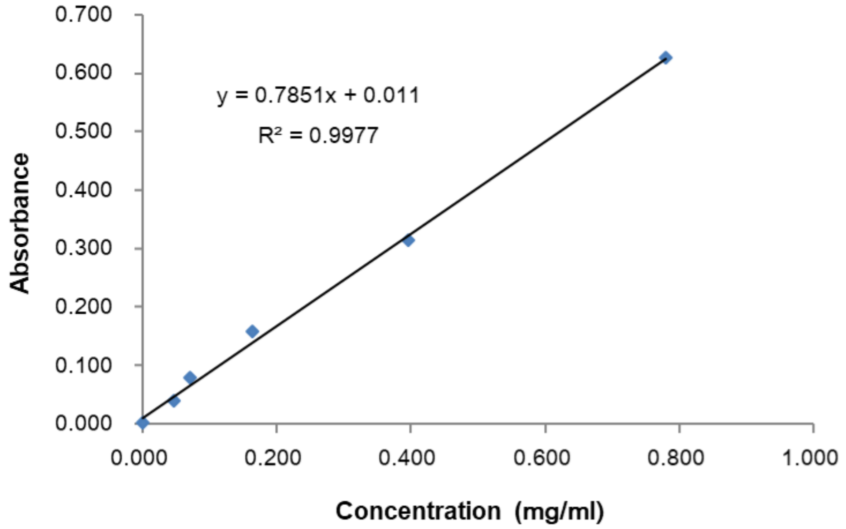

Figure S5. Standard curve for the determination of total polyphenol content. A stock solution of gallic acid at a concentration of 1.000 mg/mL was prepared in ethanol. All calibration solutions (0.001, 0.039, 0.078, 0.156, 0.313, and 0.625 mg/mL) were then prepared by serially diluting the stock solution with ethanol. Finally, the gallic acid standard curve equation was obtained: $y = 0.7851x + 0.011$, $R^2 = 0.9977$. The total phenolic content of each sample was calculated using the calibration curve, and mg gallic acid equivalent (GAE) g^{-1} FW was used to represent the polyphenol content.

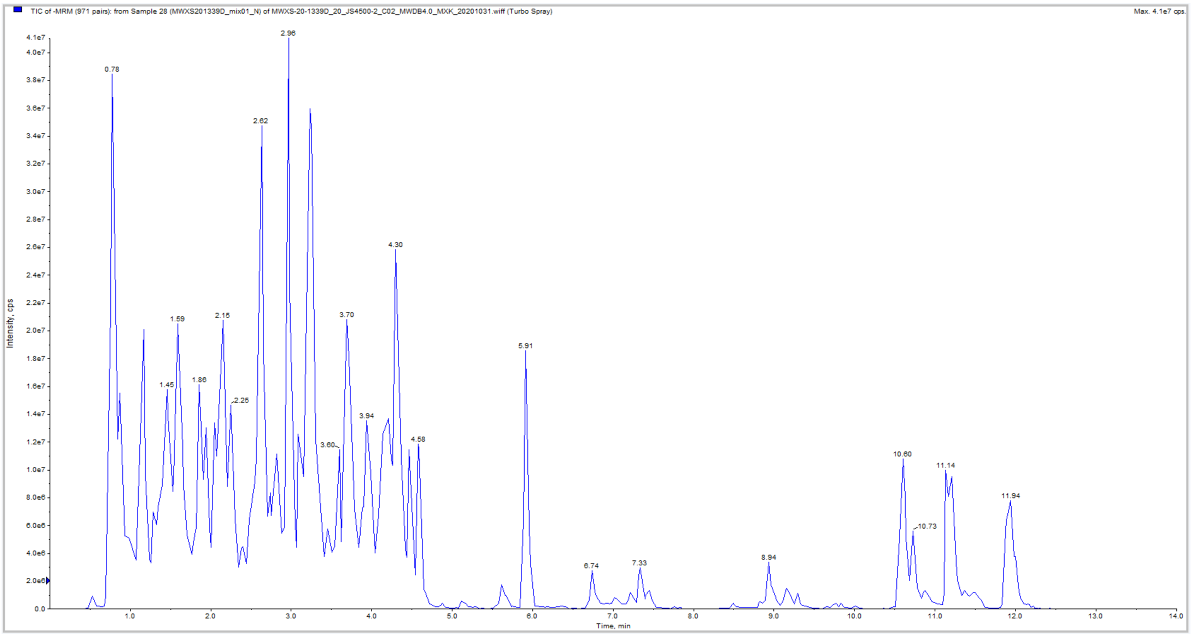

TIC-N（negative ion mode）

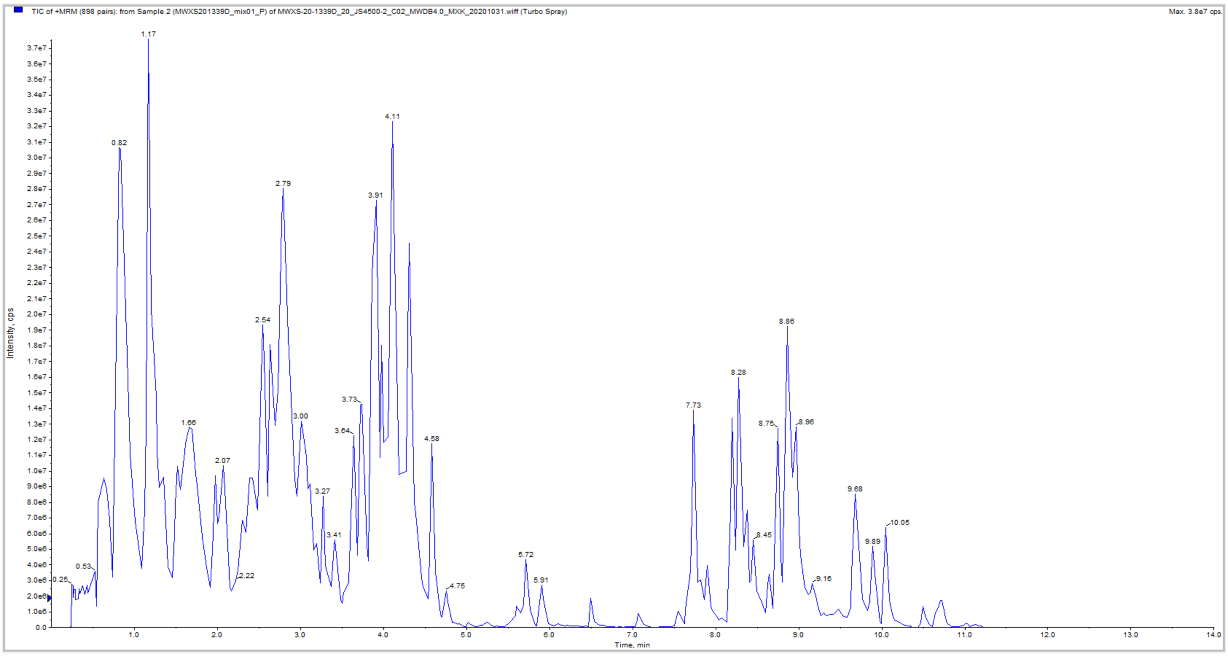

TIC-P（Positive ion mode）

Figure S6. Total ions current of endopleura and embryo samples was analyzed by mass sp ectrometry.

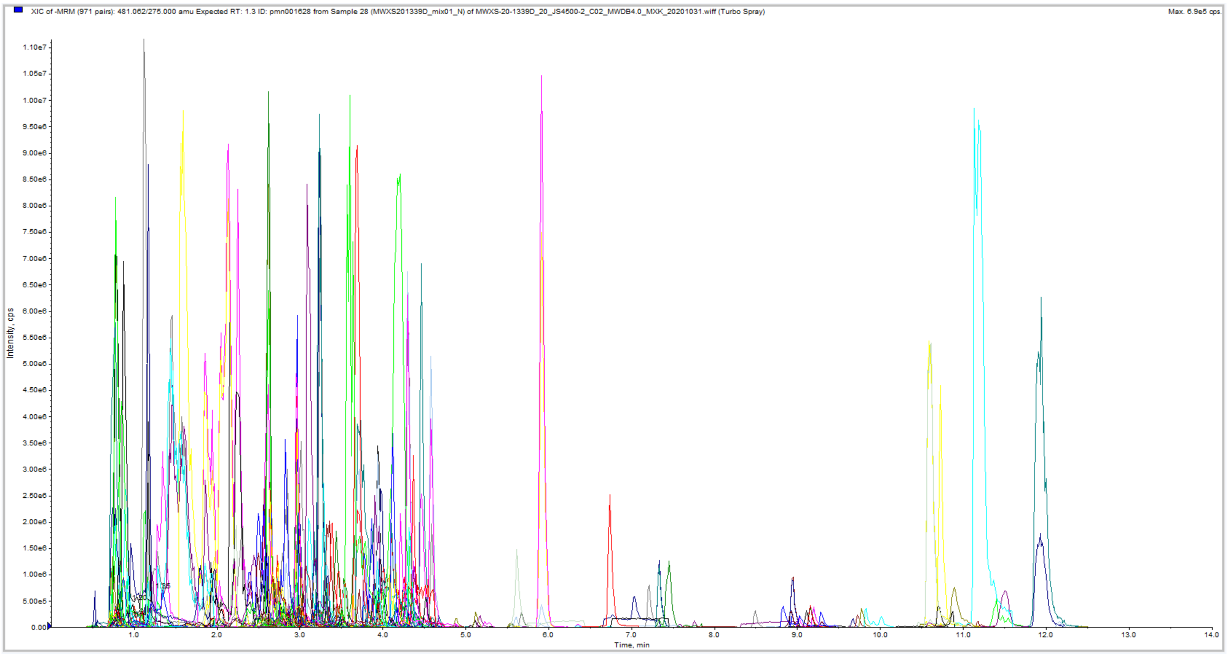

MRM-N（negative ion mode）

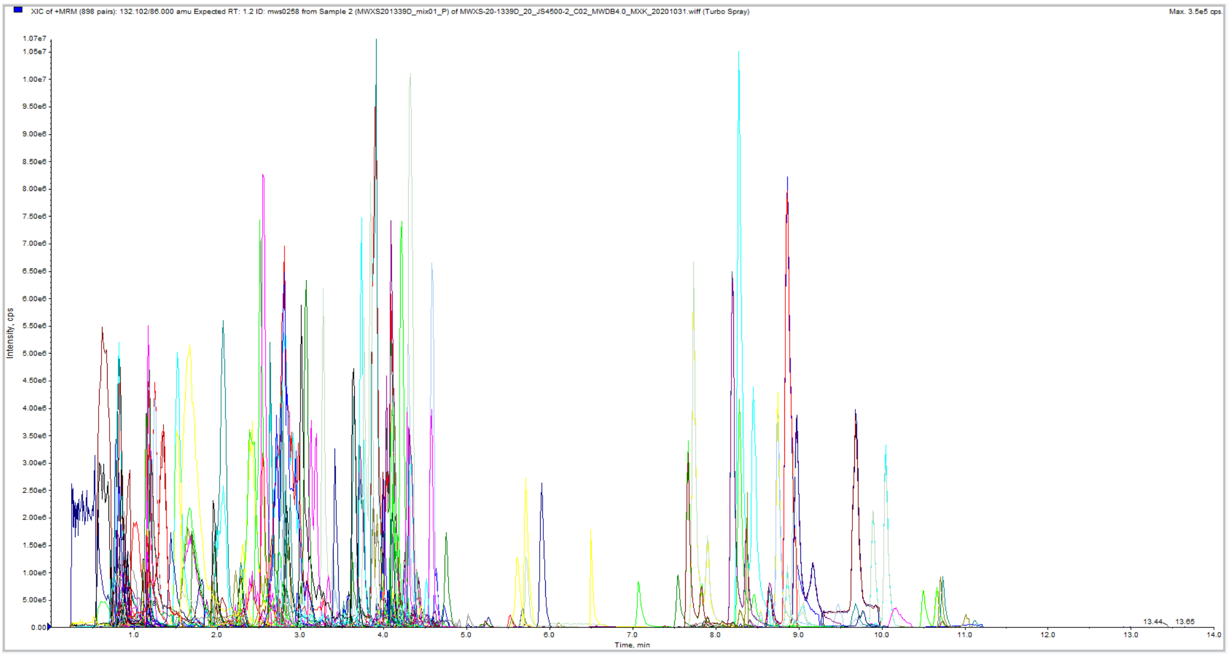

MRM-P（Positive ion mode）

Figure S7. Multi-peak diagram of sample metabolite detection.

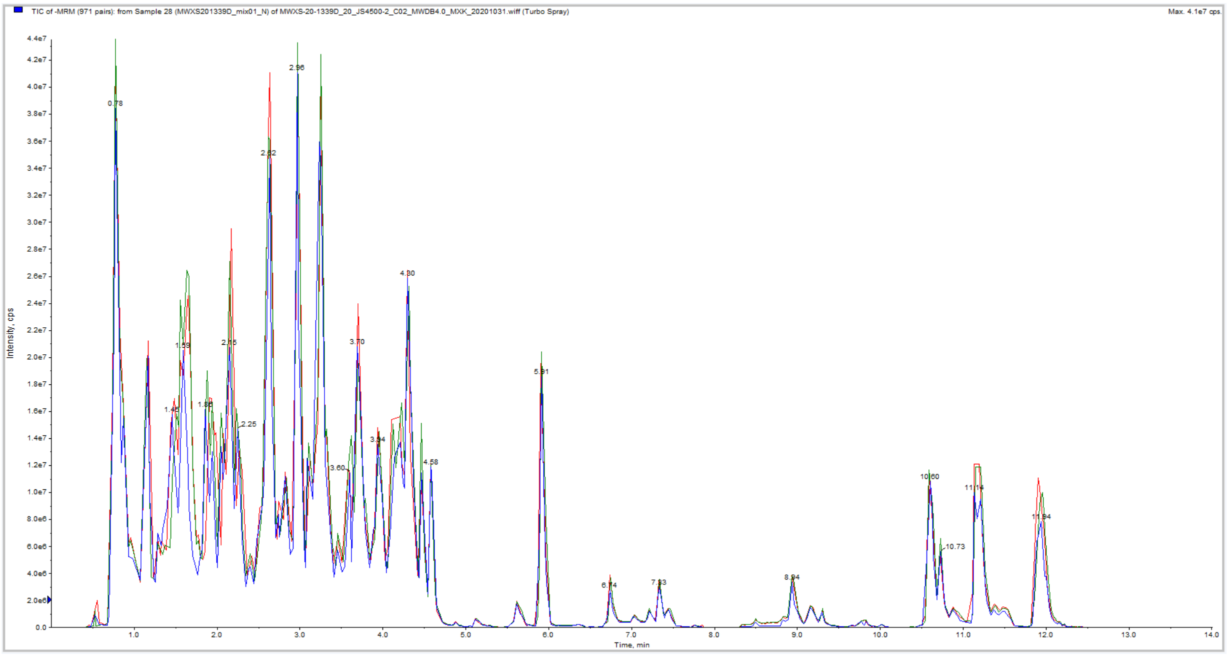

QC-MS/TIC-overlap-N（negative ion mode）

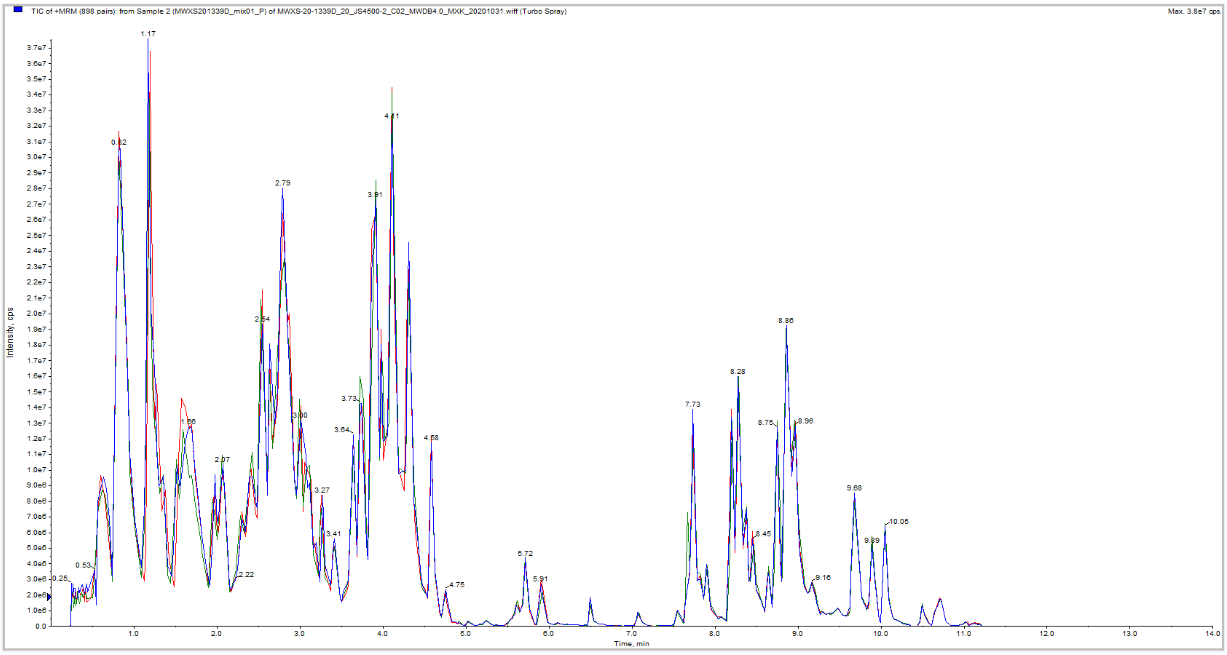

QC-MS/TIC-overlap-P（positive ion mode）

Figure S8. Quality control sample detection multi-peak diagram.
